# *catena*-Poly[[(triphenyl­phosphane)copper(I)]-di-μ-iodido-[(triphenyl­phosphane)copper(I)]-μ-{1,2-bis­[1-(pyridin-4-yl)ethyl­idene]hydrazine}]

**DOI:** 10.1107/S1600536811036555

**Published:** 2011-09-14

**Authors:** Hoong-Kun Fun, Wan-Sin Loh, Goutam K. Patra, Anindita Mukherjee, Pankaj K. Pal

**Affiliations:** a X-ray Crystallography Unit, School of Physics, Universiti Sains Malaysia, 11800 USM, Penang, Malaysia; b Department of Chemistry, Vijoygarh Jyotish Ray College, Jadavpur, Kolkata, 700 032, India

## Abstract

In the title coordination polymer, [Cu_2_I_2_(C_14_H_14_N_4_)(C_18_H_15_P)_2_]_*n*_, the Cu^I^ atom is coordinated by two I atoms, one P atom and one N atom in a fairly regular tetra­hedral arrangement. A crystallographic inversion centre generates a Cu_2_I_2_ diamond with a Cu–Cu separation of 3.0120 (5) Å. The complete *N,N*′-(1-pyridin-4-yl-ethethyl­idene)-hydrazine mol­ecule is also generated by inversion symmetry, and this bridging ligand leads to [011] polymeric chains in the crystal structure.

## Related literature

For background to copper(I) iodide and triphenyl­phosphine networks, see: Siedel & Stang (2002 ▶); Fujita *et al.* (2005 ▶); Banerjee *et al.* (2008 ▶); Zhou *et al.* (2006 ▶); Yam & Lo (1999 ▶). For the stability of the temperature controller used in the data collection, see: Cosier & Glazer (1986 ▶).
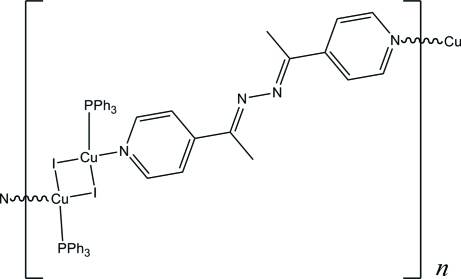


## Experimental

### 

#### Crystal data

[Cu_2_I_2_(C_14_H_14_N_4_)(C_18_H_15_P)_2_]
*M* *_r_* = 1143.72
Triclinic, 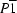

*a* = 9.2788 (4) Å
*b* = 11.4322 (5) Å
*c* = 12.4204 (5) Å
α = 74.566 (2)°
β = 76.690 (2)°
γ = 72.067 (2)°
*V* = 1192.41 (9) Å^3^
*Z* = 1
Mo *K*α radiation
μ = 2.29 mm^−1^
*T* = 100 K
0.36 × 0.23 × 0.15 mm

#### Data collection

Bruker SMART APEXII CCD diffractometer
Absorption correction: multi-scan (*SADABS*; Bruker, 2009 ▶) *T*_min_ = 0.494, *T*_max_ = 0.724
33229 measured reflections
8881 independent reflections
7865 reflections with *I* > 2σ(*I*)
*R*_int_ = 0.041

#### Refinement

*R*[*F* ^2^ > 2σ(*F* ^2^)] = 0.032
*wR*(*F* ^2^) = 0.086
*S* = 1.05
8881 reflections
272 parameters
H-atom parameters constrained
Δρ_max_ = 2.34 e Å^−3^
Δρ_min_ = −1.81 e Å^−3^

### 

Data collection: *APEX2* (Bruker, 2009 ▶); cell refinement: *SAINT* (Bruker, 2009 ▶); data reduction: *SAINT*; program(s) used to solve structure: *SHELXTL* (Sheldrick, 2008 ▶); program(s) used to refine structure: *SHELXTL*; molecular graphics: *SHELXTL*; software used to prepare material for publication: *SHELXTL* and *PLATON* (Spek, 2009 ▶).

## Supplementary Material

Crystal structure: contains datablock(s) global, I. DOI: 10.1107/S1600536811036555/hb6379sup1.cif

Structure factors: contains datablock(s) I. DOI: 10.1107/S1600536811036555/hb6379Isup2.hkl

Additional supplementary materials:  crystallographic information; 3D view; checkCIF report

## Figures and Tables

**Table:** 

Cu1—I1^i^	2.6417 (3)
Cu1—I1	2.6781 (3)
Cu1—N1	2.0586 (15)
Cu1—P1	2.2278 (5)

**Table:** 

Cu1^i^—I1—Cu1	68.967 (9)

Symmetry code: (i) 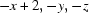.

**Fig. 1.**
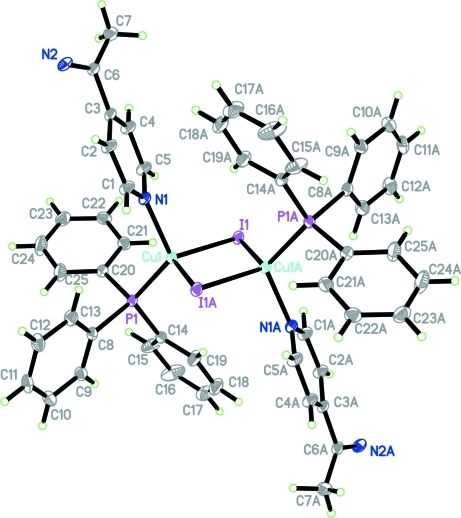
The molecular structure of the title compound, showing 50% probability displacement ellipsoids.

**Fig. 2.**
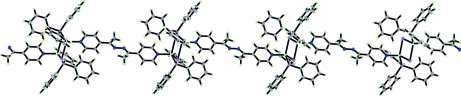
The polymeric chain of the title compound with 50% probability ellipsoids for non H atoms, showing the coordination environment for the N atoms.

**Fig. 3.**
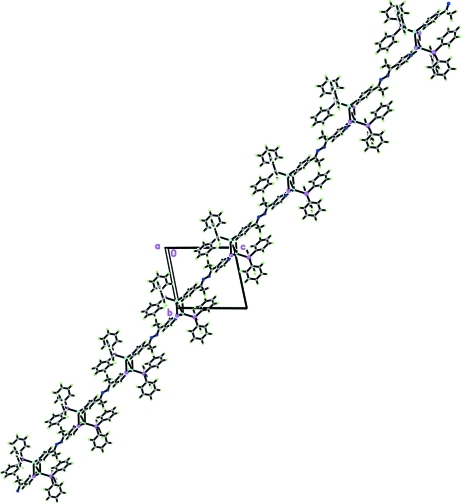
The crystal packing of the title compound, viewed along the a axis.

## supplementary crystallographic information

### Comment

Copper(I) iodides are interesting building blocks for the formation of extended solid-state coordination architectures (Siedel *et al.*, 2002; Fujita *et al.*, 2005; Banerjee *et al.*, 2008). Copper(I) complexes with PPh_3_ as a co-ligand are of rising importance owing to their diverse structures and photophysical and chemical properties (Zhou *et al.*, 2006; Yam & Lo, 1999).

The asymmetric unit of the title polymeric compound (Fig. 1) contains one Cu^I^ cation, one iodine anion, one triphenylphosphane unit and one *N,N*'-(1-pyridin-4-yl-ethethylidene)-hydrazine unit. The other half being generated by an inversion center (symmetry code: -*x* + 2, -*y*, -*z*). Each Cu^I^ cation is tetracoordinated by one nitrogen, one phosphorus and two iodine atoms. The Cu–I distances are 2.6417 (3) and 2.6781 (3) Å. In the crystal (Fig. 2 & Fig. 3), the nitrogen atoms are bridged together, leading to the formation of polymeric chains along the [011].

### Experimental

The *N,N*'-bis-(1-pyridin-4-yl-ethethylidene)-hydrazine component was prepared in good yield as a yellow solid by condensing hydrazine hydrate with 4-acetylpyridine in anhydrous methanol in 1:2 molar ratio. The Cu(I) complex was prepared in the following way: to a solution of PPh_3_ (0.262 g, 1 mmol) in CH_3_CN (50 ml), CuI (0.19 g, 1 mmol) was added. The reaction mixture was stirred for about 1 h to obtain a white turbid solution. Then *N,N*'-bis-(1-pyridin-4-yl-ethethylidene)-hydrazine (0.238 g, 1 mmol) in 20 ml CHCl_3_ was added with constant stirring at room temperature to give a clear yellowish solution. Orange–red block-shaped crystals were obtained by slow evaporation of the solution after 2 days. Yield: 0.45 g (70%).

### Refinement

All H atoms were positioned geometrically and refined using a riding model with *U*_iso_(H) = 1.2 *U*_eq_(C) [C–H = 0.95–0.98 Å].

### Crystal data

**Table:**

[Cu_2_I_2_(C_14_H_14_N_4_)(C_18_H_15_P)_2_]	*Z* = 1
*M**_r_* = 1143.72	*F*(000) = 566
Triclinic, *P*1	*D*_x_ = 1.593 Mg m^−^^3^
Hall symbol: -P 1	Mo *K*α radiation, λ = 0.71073 Å
*a* = 9.2788 (4) Å	Cell parameters from 9948 reflections
*b* = 11.4322 (5) Å	θ = 2.3–34.9°
*c* = 12.4204 (5) Å	µ = 2.29 mm^−^^1^
α = 74.566 (2)°	*T* = 100 K
β = 76.690 (2)°	Block, orange
γ = 72.067 (2)°	0.36 × 0.23 × 0.15 mm
*V* = 1192.41 (9) Å^3^	

### Data collection

**Table:**

Bruker SMART APEXII CCD diffractometer	8881 independent reflections
Radiation source: fine-focus sealed tube	7865 reflections with *I* > 2σ(*I*)
graphite	*R*_int_ = 0.041
φ and ω scans	θ_max_ = 33.0°, θ_min_ = 1.7°
Absorption correction: multi-scan (*SADABS*; Bruker, 2009)	*h* = −14→14
*T*_min_ = 0.494, *T*_max_ = 0.724	*k* = −17→16
33229 measured reflections	*l* = −18→19

### Refinement

**Table:**

Refinement on *F*^2^	Primary atom site location: structure-invariant direct methods
Least-squares matrix: full	Secondary atom site location: difference Fourier map
*R*[*F*^2^ > 2σ(*F*^2^)] = 0.032	Hydrogen site location: inferred from neighbouring sites
*wR*(*F*^2^) = 0.086	H-atom parameters constrained
*S* = 1.05	*w* = 1/[σ^2^(*F*_o_^2^) + (0.0516*P*)^2^ + 0.4005*P*] where *P* = (*F*_o_^2^ + 2*F*_c_^2^)/3
8881 reflections	(Δ/σ)_max_ = 0.002
272 parameters	Δρ_max_ = 2.34 e Å^−^^3^
0 restraints	Δρ_min_ = −1.81 e Å^−^^3^

### Special details

**Table:**

Experimental. The crystal was placed in the cold stream of an Oxford Cryosystems Cobra open-flow nitrogen cryostat (Cosier & Glazer, 1986) operating at 100.0 (1) K.
Geometry. All e.s.d.'s (except the e.s.d. in the dihedral angle between two l.s. planes) are estimated using the full covariance matrix. The cell e.s.d.'s are taken into account individually in the estimation of e.s.d.'s in distances, angles and torsion angles; correlations between e.s.d.'s in cell parameters are only used when they are defined by crystal symmetry. An approximate (isotropic) treatment of cell e.s.d.'s is used for estimating e.s.d.'s involving l.s. planes.
Refinement. Refinement of *F*^2^ against ALL reflections. The weighted *R*-factor *wR* and goodness of fit *S* are based on *F*^2^, conventional *R*-factors *R* are based on *F*, with *F* set to zero for negative *F*^2^. The threshold expression of *F*^2^ > σ(*F*^2^) is used only for calculating *R*-factors(gt) *etc*. and is not relevant to the choice of reflections for refinement. *R*-factors based on *F*^2^ are statistically about twice as large as those based on *F*, and *R*- factors based on ALL data will be even larger.

### Fractional atomic coordinates and isotropic or equivalent isotropic displacement parameters (Å^2^)

**Table:**

	*x*	*y*	*z*	*U*_iso_*/*U*_eq_	
I1	0.855859 (12)	−0.132037 (10)	0.055940 (9)	0.01592 (4)	
Cu1	0.86308 (2)	0.10732 (2)	0.023918 (18)	0.01484 (5)	
P1	0.75620 (5)	0.16954 (4)	0.18626 (4)	0.01378 (8)	
N1	0.74367 (17)	0.19616 (15)	−0.10892 (13)	0.0163 (3)	
N2	0.53664 (18)	0.47410 (16)	−0.45218 (13)	0.0191 (3)	
C1	0.7889 (2)	0.28666 (19)	−0.19227 (16)	0.0201 (3)	
H1A	0.8756	0.3112	−0.1869	0.024*	
C2	0.7158 (2)	0.34564 (19)	−0.28518 (16)	0.0203 (3)	
H2A	0.7511	0.4099	−0.3412	0.024*	
C3	0.5898 (2)	0.31012 (17)	−0.29597 (15)	0.0160 (3)	
C4	0.5405 (2)	0.21851 (19)	−0.20863 (16)	0.0201 (3)	
H4A	0.4539	0.1924	−0.2115	0.024*	
C5	0.6193 (2)	0.16582 (19)	−0.11742 (16)	0.0196 (3)	
H5A	0.5828	0.1049	−0.0578	0.024*	
C6	0.5135 (2)	0.36689 (18)	−0.39770 (15)	0.0171 (3)	
C7	0.4228 (3)	0.2953 (2)	−0.42808 (18)	0.0248 (4)	
H7A	0.3972	0.3349	−0.5040	0.037*	
H7B	0.3280	0.2960	−0.3732	0.037*	
H7C	0.4839	0.2082	−0.4269	0.037*	
C8	0.79470 (19)	0.30849 (17)	0.20650 (15)	0.0153 (3)	
C9	0.8041 (2)	0.32434 (19)	0.31231 (16)	0.0187 (3)	
H9A	0.7892	0.2611	0.3780	0.022*	
C10	0.8351 (2)	0.4324 (2)	0.32155 (18)	0.0227 (4)	
H10A	0.8428	0.4422	0.3935	0.027*	
C11	0.8550 (2)	0.52586 (19)	0.22673 (19)	0.0236 (4)	
H11A	0.8755	0.5997	0.2338	0.028*	
C12	0.8450 (3)	0.5120 (2)	0.12104 (18)	0.0247 (4)	
H12A	0.8580	0.5762	0.0559	0.030*	
C13	0.8158 (2)	0.40299 (19)	0.11159 (17)	0.0223 (4)	
H13A	0.8102	0.3929	0.0393	0.027*	
C14	0.8022 (2)	0.05253 (17)	0.31409 (15)	0.0152 (3)	
C15	0.6953 (2)	0.0328 (2)	0.41092 (18)	0.0290 (5)	
H15A	0.5914	0.0808	0.4125	0.035*	
C16	0.7404 (3)	−0.0575 (3)	0.5063 (2)	0.0391 (6)	
H16A	0.6667	−0.0713	0.5721	0.047*	
C17	0.8924 (3)	−0.1270 (2)	0.5051 (2)	0.0321 (5)	
H17A	0.9229	−0.1884	0.5699	0.038*	
C18	0.9990 (3)	−0.1063 (2)	0.4092 (2)	0.0332 (5)	
H18A	1.1036	−0.1523	0.4087	0.040*	
C19	0.9544 (2)	−0.0186 (2)	0.31328 (17)	0.0250 (4)	
H19A	1.0279	−0.0070	0.2468	0.030*	
C20	0.5469 (2)	0.21052 (18)	0.20193 (16)	0.0173 (3)	
C21	0.4819 (2)	0.1214 (2)	0.18810 (17)	0.0201 (3)	
H21A	0.5455	0.0406	0.1792	0.024*	
C22	0.3255 (2)	0.1495 (2)	0.18721 (17)	0.0220 (4)	
H22A	0.2825	0.0876	0.1788	0.026*	
C23	0.2321 (2)	0.2676 (2)	0.1986 (2)	0.0267 (4)	
H23A	0.1256	0.2879	0.1957	0.032*	
C24	0.2950 (3)	0.3556 (2)	0.2141 (3)	0.0363 (6)	
H24A	0.2308	0.4362	0.2232	0.044*	
C25	0.4523 (2)	0.3274 (2)	0.2166 (2)	0.0296 (5)	
H25A	0.4942	0.3882	0.2284	0.036*	

### Atomic displacement parameters (Å^2^)

**Table:**

	*U*^11^	*U*^22^	*U*^33^	*U*^12^	*U*^13^	*U*^23^
I1	0.01722 (6)	0.01009 (6)	0.01945 (6)	−0.00324 (4)	−0.00483 (4)	−0.00023 (4)
Cu1	0.01887 (10)	0.01192 (11)	0.01240 (10)	−0.00298 (8)	−0.00424 (7)	−0.00015 (7)
P1	0.01561 (18)	0.0109 (2)	0.01386 (19)	−0.00234 (14)	−0.00335 (14)	−0.00154 (15)
N1	0.0198 (6)	0.0119 (7)	0.0153 (7)	−0.0020 (5)	−0.0060 (5)	0.0005 (5)
N2	0.0205 (7)	0.0176 (8)	0.0159 (7)	−0.0021 (6)	−0.0077 (5)	0.0028 (6)
C1	0.0239 (8)	0.0159 (9)	0.0201 (8)	−0.0071 (7)	−0.0094 (7)	0.0042 (6)
C2	0.0240 (8)	0.0167 (9)	0.0193 (8)	−0.0065 (7)	−0.0098 (7)	0.0046 (6)
C3	0.0175 (7)	0.0127 (8)	0.0154 (7)	−0.0002 (6)	−0.0054 (6)	−0.0008 (6)
C4	0.0209 (8)	0.0187 (9)	0.0196 (8)	−0.0063 (6)	−0.0074 (6)	0.0025 (6)
C5	0.0218 (8)	0.0185 (9)	0.0161 (8)	−0.0064 (6)	−0.0062 (6)	0.0046 (6)
C6	0.0185 (7)	0.0150 (8)	0.0150 (7)	0.0000 (6)	−0.0052 (6)	−0.0014 (6)
C7	0.0348 (10)	0.0171 (9)	0.0245 (9)	−0.0056 (8)	−0.0146 (8)	−0.0007 (7)
C8	0.0148 (7)	0.0115 (8)	0.0182 (8)	−0.0022 (5)	−0.0028 (6)	−0.0021 (6)
C9	0.0206 (8)	0.0159 (8)	0.0197 (8)	−0.0047 (6)	−0.0033 (6)	−0.0039 (6)
C10	0.0264 (9)	0.0198 (9)	0.0238 (9)	−0.0062 (7)	−0.0032 (7)	−0.0085 (7)
C11	0.0234 (8)	0.0136 (9)	0.0329 (11)	−0.0046 (7)	0.0000 (7)	−0.0074 (7)
C12	0.0319 (10)	0.0144 (9)	0.0253 (10)	−0.0067 (7)	−0.0020 (8)	−0.0013 (7)
C13	0.0293 (9)	0.0154 (9)	0.0201 (9)	−0.0050 (7)	−0.0054 (7)	−0.0002 (7)
C14	0.0186 (7)	0.0125 (8)	0.0141 (7)	−0.0040 (6)	−0.0038 (6)	−0.0011 (6)
C15	0.0210 (8)	0.0424 (14)	0.0192 (9)	−0.0099 (8)	−0.0056 (7)	0.0044 (8)
C16	0.0348 (11)	0.0576 (18)	0.0215 (10)	−0.0234 (12)	−0.0088 (9)	0.0141 (10)
C17	0.0445 (12)	0.0253 (11)	0.0257 (10)	−0.0109 (9)	−0.0178 (9)	0.0083 (8)
C18	0.0364 (11)	0.0292 (12)	0.0237 (10)	0.0098 (9)	−0.0124 (9)	−0.0035 (8)
C19	0.0234 (8)	0.0260 (11)	0.0171 (8)	0.0041 (7)	−0.0038 (7)	−0.0022 (7)
C20	0.0173 (7)	0.0158 (8)	0.0176 (8)	−0.0017 (6)	−0.0047 (6)	−0.0033 (6)
C21	0.0189 (7)	0.0213 (9)	0.0210 (9)	−0.0047 (6)	−0.0026 (6)	−0.0072 (7)
C22	0.0192 (8)	0.0273 (10)	0.0228 (9)	−0.0084 (7)	−0.0035 (7)	−0.0080 (7)
C23	0.0179 (8)	0.0292 (11)	0.0329 (11)	−0.0035 (7)	−0.0089 (7)	−0.0057 (8)
C24	0.0201 (9)	0.0222 (11)	0.0659 (18)	0.0019 (8)	−0.0124 (10)	−0.0121 (11)
C25	0.0197 (8)	0.0194 (10)	0.0517 (14)	−0.0007 (7)	−0.0104 (9)	−0.0120 (9)

### Geometric parameters (Å, °)

**Table:**

Cu1—I1^i^	2.6417 (3)	C10—C11	1.383 (3)
Cu1—I1	2.6781 (3)	C10—H10A	0.9500
Cu1—N1	2.0586 (15)	C11—C12	1.390 (3)
Cu1—P1	2.2278 (5)	C11—H11A	0.9500
Cu1—I1^i^	2.6417 (3)	C12—C13	1.393 (3)
Cu1—Cu1^i^	3.0120 (5)	C12—H12A	0.9500
P1—C8	1.8214 (19)	C13—H13A	0.9500
P1—C14	1.8228 (18)	C14—C15	1.387 (3)
P1—C20	1.8288 (18)	C14—C19	1.396 (3)
N1—C5	1.336 (2)	C15—C16	1.398 (3)
N1—C1	1.348 (2)	C15—H15A	0.9500
N2—C6	1.288 (2)	C16—C17	1.389 (4)
N2—N2^ii^	1.405 (3)	C16—H16A	0.9500
C1—C2	1.383 (2)	C17—C18	1.380 (4)
C1—H1A	0.9500	C17—H17A	0.9500
C2—C3	1.394 (3)	C18—C19	1.390 (3)
C2—H2A	0.9500	C18—H18A	0.9500
C3—C4	1.395 (3)	C19—H19A	0.9500
C3—C6	1.487 (2)	C20—C25	1.386 (3)
C4—C5	1.388 (2)	C20—C21	1.396 (3)
C4—H4A	0.9500	C21—C22	1.389 (3)
C5—H5A	0.9500	C21—H21A	0.9500
C6—C7	1.503 (3)	C22—C23	1.385 (3)
C7—H7A	0.9800	C22—H22A	0.9500
C7—H7B	0.9800	C23—C24	1.381 (3)
C7—H7C	0.9800	C23—H23A	0.9500
C8—C13	1.393 (3)	C24—C25	1.401 (3)
C8—C9	1.399 (3)	C24—H24A	0.9500
C9—C10	1.390 (3)	C25—H25A	0.9500
C9—H9A	0.9500		
			
Cu1^i^—I1—Cu1	68.967 (9)	C8—C9—H9A	119.9
N1—Cu1—P1	115.34 (5)	C11—C10—C9	120.45 (19)
N1—Cu1—I1^i^	104.66 (5)	C11—C10—H10A	119.8
P1—Cu1—I1^i^	115.133 (15)	C9—C10—H10A	119.8
N1—Cu1—I1	102.74 (5)	C10—C11—C12	120.15 (19)
P1—Cu1—I1	107.279 (15)	C10—C11—H11A	119.9
I1^i^—Cu1—I1	111.033 (9)	C12—C11—H11A	119.9
N1—Cu1—Cu1^i^	114.71 (5)	C11—C12—C13	119.39 (19)
P1—Cu1—Cu1^i^	129.538 (16)	C11—C12—H12A	120.3
I1^i^—Cu1—Cu1^i^	56.087 (8)	C13—C12—H12A	120.3
I1—Cu1—Cu1^i^	54.946 (8)	C12—C13—C8	121.04 (19)
C8—P1—C14	103.50 (8)	C12—C13—H13A	119.5
C8—P1—C20	103.58 (8)	C8—C13—H13A	119.5
C14—P1—C20	104.71 (8)	C15—C14—C19	119.24 (17)
C8—P1—Cu1	117.67 (6)	C15—C14—P1	123.60 (14)
C14—P1—Cu1	115.48 (6)	C19—C14—P1	117.15 (14)
C20—P1—Cu1	110.48 (6)	C14—C15—C16	120.1 (2)
C5—N1—C1	116.93 (15)	C14—C15—H15A	120.0
C5—N1—Cu1	121.74 (12)	C16—C15—H15A	120.0
C1—N1—Cu1	121.32 (12)	C17—C16—C15	120.3 (2)
C6—N2—N2^ii^	113.4 (2)	C17—C16—H16A	119.9
N1—C1—C2	123.36 (18)	C15—C16—H16A	119.9
N1—C1—H1A	118.3	C18—C17—C16	119.6 (2)
C2—C1—H1A	118.3	C18—C17—H17A	120.2
C1—C2—C3	119.49 (17)	C16—C17—H17A	120.2
C1—C2—H2A	120.3	C17—C18—C19	120.4 (2)
C3—C2—H2A	120.3	C17—C18—H18A	119.8
C2—C3—C4	117.29 (16)	C19—C18—H18A	119.8
C2—C3—C6	120.86 (17)	C18—C19—C14	120.3 (2)
C4—C3—C6	121.85 (17)	C18—C19—H19A	119.8
C5—C4—C3	119.33 (17)	C14—C19—H19A	119.8
C5—C4—H4A	120.3	C25—C20—C21	118.99 (17)
C3—C4—H4A	120.3	C25—C20—P1	123.81 (16)
N1—C5—C4	123.53 (17)	C21—C20—P1	116.96 (14)
N1—C5—H5A	118.2	C22—C21—C20	120.79 (19)
C4—C5—H5A	118.2	C22—C21—H21A	119.6
N2—C6—C3	114.47 (17)	C20—C21—H21A	119.6
N2—C6—C7	126.95 (17)	C23—C22—C21	120.06 (19)
C3—C6—C7	118.56 (17)	C23—C22—H22A	120.0
C6—C7—H7A	109.5	C21—C22—H22A	120.0
C6—C7—H7B	109.5	C24—C23—C22	119.50 (18)
H7A—C7—H7B	109.5	C24—C23—H23A	120.2
C6—C7—H7C	109.5	C22—C23—H23A	120.2
H7A—C7—H7C	109.5	C23—C24—C25	120.7 (2)
H7B—C7—H7C	109.5	C23—C24—H24A	119.6
C13—C8—C9	118.83 (18)	C25—C24—H24A	119.6
C13—C8—P1	118.03 (14)	C20—C25—C24	119.9 (2)
C9—C8—P1	123.14 (14)	C20—C25—H25A	120.1
C10—C9—C8	120.13 (18)	C24—C25—H25A	120.1
C10—C9—H9A	119.9		
			
Cu1^i^—I1—Cu1—N1	111.41 (5)	Cu1—P1—C8—C13	32.33 (16)
Cu1^i^—I1—Cu1—P1	−126.595 (17)	C14—P1—C8—C9	−18.90 (17)
Cu1^i^—I1—Cu1—I1^i^	0.0	C20—P1—C8—C9	90.17 (16)
N1—Cu1—P1—C8	−84.49 (8)	Cu1—P1—C8—C9	−147.61 (13)
I1^i^—Cu1—P1—C8	37.63 (6)	C13—C8—C9—C10	−0.5 (3)
I1—Cu1—P1—C8	161.76 (6)	P1—C8—C9—C10	179.45 (15)
Cu1^i^—Cu1—P1—C8	103.30 (6)	C8—C9—C10—C11	0.9 (3)
N1—Cu1—P1—C14	152.70 (8)	C9—C10—C11—C12	−0.4 (3)
I1^i^—Cu1—P1—C14	−85.18 (7)	C10—C11—C12—C13	−0.4 (3)
I1—Cu1—P1—C14	38.95 (7)	C11—C12—C13—C8	0.8 (3)
Cu1^i^—Cu1—P1—C14	−19.51 (7)	C9—C8—C13—C12	−0.3 (3)
N1—Cu1—P1—C20	34.12 (9)	P1—C8—C13—C12	179.73 (16)
I1^i^—Cu1—P1—C20	156.24 (7)	C8—P1—C14—C15	91.61 (19)
I1—Cu1—P1—C20	−79.63 (7)	C20—P1—C14—C15	−16.6 (2)
Cu1^i^—Cu1—P1—C20	−138.08 (7)	Cu1—P1—C14—C15	−138.34 (17)
P1—Cu1—N1—C5	−77.61 (16)	C8—P1—C14—C19	−87.26 (17)
I1^i^—Cu1—N1—C5	154.82 (14)	C20—P1—C14—C19	164.52 (16)
I1—Cu1—N1—C5	38.75 (16)	Cu1—P1—C14—C19	42.79 (18)
Cu1^i^—Cu1—N1—C5	95.78 (15)	C19—C14—C15—C16	−0.1 (4)
P1—Cu1—N1—C1	103.31 (15)	P1—C14—C15—C16	−178.9 (2)
I1^i^—Cu1—N1—C1	−24.26 (16)	C14—C15—C16—C17	0.7 (4)
I1—Cu1—N1—C1	−140.33 (15)	C15—C16—C17—C18	0.1 (4)
Cu1^i^—Cu1—N1—C1	−83.30 (16)	C16—C17—C18—C19	−1.5 (4)
C5—N1—C1—C2	−1.6 (3)	C17—C18—C19—C14	2.1 (4)
Cu1—N1—C1—C2	177.55 (16)	C15—C14—C19—C18	−1.3 (3)
N1—C1—C2—C3	−1.0 (3)	P1—C14—C19—C18	177.6 (2)
C1—C2—C3—C4	2.3 (3)	C8—P1—C20—C25	4.4 (2)
C1—C2—C3—C6	−176.79 (18)	C14—P1—C20—C25	112.59 (19)
C2—C3—C4—C5	−1.2 (3)	Cu1—P1—C20—C25	−122.45 (18)
C6—C3—C4—C5	177.88 (19)	C8—P1—C20—C21	178.64 (15)
C1—N1—C5—C4	2.8 (3)	C14—P1—C20—C21	−73.20 (16)
Cu1—N1—C5—C4	−176.36 (16)	Cu1—P1—C20—C21	51.76 (16)
C3—C4—C5—N1	−1.4 (3)	C25—C20—C21—C22	1.0 (3)
N2^ii^—N2—C6—C3	−179.94 (18)	P1—C20—C21—C22	−173.48 (16)
N2^ii^—N2—C6—C7	1.5 (3)	C20—C21—C22—C23	0.9 (3)
C2—C3—C6—N2	−21.8 (3)	C21—C22—C23—C24	−1.9 (3)
C4—C3—C6—N2	159.10 (19)	C22—C23—C24—C25	1.0 (4)
C2—C3—C6—C7	156.82 (19)	C21—C20—C25—C24	−1.9 (3)
C4—C3—C6—C7	−22.2 (3)	P1—C20—C25—C24	172.2 (2)
C14—P1—C8—C13	161.04 (15)	C23—C24—C25—C20	0.9 (4)
C20—P1—C8—C13	−89.89 (16)		

Symmetry codes: (i) −*x*+2, −*y*, −*z*; (ii) −*x*+1, −*y*+1, −*z*−1.
